# Initial timing of alfalfa hay supplementation manipulates blood parameters, rumen gene expression, and epithelial microbiota in pre-weaning lambs

**DOI:** 10.1093/jas/skae188

**Published:** 2024-07-20

**Authors:** Kenan Li, Haidong Du, Wenliang Guo, Meila Na, Renhua Na

**Affiliations:** College of Animal Science, Inner Mongolia Agricultural University, Hohhot, China; College of Animal Science, Inner Mongolia Agricultural University, Hohhot, China; College of Animal Science, Inner Mongolia Agricultural University, Hohhot, China; College of Animal Science, Inner Mongolia Agricultural University, Hohhot, China; College of Animal Science, Inner Mongolia Agricultural University, Hohhot, China

**Keywords:** growth, alfalfa hay supplementation, pre-weaning lambs, rumen epithelial microbiota

## Abstract

The present study aimed to investigate the impact of initiating alfalfa supplementation at either 14 d or 42 d of age on growth performance, blood parameters, rumen tissue gene expression, and epithelial microbiota in pre-weaning lambs. A total of 42 seven-day-old male Hu lambs (3.88 ± 0.92 kg) were selected for this study. After 7 d of adjustment period, 6 lambs were slaughtered at 14 d of age to establish a baseline control. The remaining 36 lambs were randomly allocated to 2 treatment groups, every 3 lambs were considered a unit, including fed milk replacer, starter pellets, and either alfalfa hay fed at 14 (**EAF**) or 42 d of age (**LAF**). Body weight and feed intake were recorded for lamb until 70 d of age. Blood samples, rumen tissue samples, and epithelial microbiota samples were collected from the lambs at 42, 56, and 70 d of age. The results indicated that average daily gain, starter intake, and total dry matter intake were greater in the EAF group compared to the LAF group from 14 to 42 d of age (*P* < 0.01), but no significant differences from 43 to 70 d of age or during the entire trial. Treatment and age interactively affected the alfalfa intake (*P* = 0.02) from 43 to 70 d of age. The concentration of serum immunoglobulin A (**IgA**) (*P* < 0.01) and the expression of the rumen gene *insulin-like growth factor-1* (*P* < 0.01) were greater in the EAF group compared to the LAF group at 42 d of age. Furthermore, the concentrations of alkaline phosphatase (*P* = 0.03), albumin (*P* < 0.01), total protein (*P* = 0.03), urea (*P* = 0.04), lipopolysaccharide (*P* < 0.01), β-hydroxybutyric acid (*P* = 0.02), interleukin-1β (**IL-1β**) (*P* < 0.01), IL-4 (*P* < 0.01), and tumor necrosis factor-α (*P* < 0.01) were affected by age. The abundance of *Prevotella* was lower (*P* < 0.05), whereas *Megasphaera* (*P* < 0.05) was greater in the EAF group compared to the LAF group at 42 d of age. The early addition of alfalfa promotes rumen epithelial microbiota colonization. In conclusion, this study demonstrated that alfalfa provision at 14 d of age promotes growth performance in lambs, but this effect disappeared at 43 to 70 d of age. Moreover, provision of alfalfa at 14 d of age enhances the immune response, promotes rumen tissue cell proliferation, and affects dynamical changes of rumen epithelial microbiota. Meanwhile, our findings showed that the rumen undergoes significant physiological challenges during the transition from a liquid diet to a solid diet.

## Teaser

Compared with providing alfalfa hay at 42 d of age, initiating the supplementation of alfalfa at 14 d of age could enhance growth performance and promote rumen development in pre-weaning lambs. Furthermore, the rumen undergoes significant physiological challenges during the transition from a liquid diet to a solid diet.

## Introduction

For young ruminants, the smooth transition from a pre-ruminant to a fully functional ruminant is an important physiological challenge (Jiao et al., 2015). This transition involves several anatomical, functional, and microbial changes in the rumen, which are primarily affected by the intake and composition of supplemental grain in young ruminants (Wu et al., 2017). Supplemental grain can rapidly ferment in the rumen and result in the production of a large amount of volatile fatty acids (**VFA**). Among these VFAs, butyrate exerts the most stimulatory effect on rumen development, with propionate following closely behind (Carroll and Hungate, 1954). However, feeding pre-weaning ruminants with only concentrates can easily lead to symptoms such as decreased rumen pH, rumen plaque formation, and keratinization of the rumen papillae (Nocek and Kesler, 1980; Beharka et al., 1998; Yang et al., 2015). In contrast, hay possesses the characteristics of coarseness, bulkiness, abrasiveness, and low energy concentration (Hill et al., 2008; Wu et al., 2017). Consequently, hay promotes chewing and rumination, 2 important activities that stimulate saliva production and maintain the appropriate pH of the rumen fluid (van Ackeren et al., 2009; Nemati et al., 2016). However, there is still controversy regarding the impact of the supplemental hay before weaning on the growth performance of ruminants. Hill et al. (2008) reported that dietary inclusion of 2.5% to 5% chopped mixed hay, which contained an average of 14.2% crude protein (**CP**), 29.5% acid detergent fiber (**ADF**), and 46.6% neutral detergent fibers (**NDF**), or 5% to 10% cottonseed hulls, with an average composition of 3.4% CP, 57.3% ADF, and 79.2% NDF, during the pre-weaning period resulted in a decrease in average daily gain (**ADG**) and dry matter intake (**DMI**). In contrast, Castells et al. (2012) found that supplementing calves with oat hay, barley straw, and triticale silage improved starter intake, ADG, and final weight, while similar results were not observed with alfalfa hay supplementation. On the one hand, newborn ruminants, due to their pseudo-monogastric nature, possess an undeveloped rumen at the age of 2 wk (Davis et al., 1998). A study has reported that the microbial communities responsible for fiber degradation in the rumen of pre-weaning ruminants are not fully established, and the accumulation of undigested roughage in the rumen may subsequently reduce their voluntary intake of concentrate (Drackley, 2008). On the other hand, an increasing number of research studies have found that feeding roughage to pre-weaned ruminants enhances rumen volume (Tamate et al., 1962), weight (Khan et al., 2011), as well as wall thickness and papillae length (Blottiere et al., 2003). Khan et al. (2011) and Castells et al. (2013) found that the provision of roughage to pre-weaning calves improved the rumen fermentation environment and solid feed intake. In addition, hay consumption may lead to reduced rumen plaque formation (Yang et al., 2015), and decreased non-nutritive oral behavior (Horvath and Miller-Cushon, 2017). Non-nutritive oral behaviors, including sucking pen fixtures and cross-sucking, are thought to have a negative impact on calf health (Babu et al., 2004). Therefore, most researchers agree that hay supplementation serves as an effective strategy for enhancing growth and rumen development in pre-weaned ruminants. Nevertheless, the feeding effect is related to multiple factors, including the sources of roughage (Movahedi et al., 2016), intake levels (Nemati et al., 2016), physical form of starter feed, feeding method of both solid and liquid feed (Xiao et al., 2020), levels of NDF in the starter pellets (Xie et al., 2020), and ratio of starch and NDF in starter (Zhao et al., 2023). It is generally believed that calves fed 10% (on a DM basis) high-quality hay (such as alfalfa and oats hay) might improve growth performance during the pre-weaning period (Imani et al., 2017). However, supplementation of low-quality hay (straw) in young ruminants can cause ruminal papilla and abomasum damage (Webb et al., 2013). Compared to straw (CP, NDF, and ADF levels of 4.2%, 74.0%, and 42.5%, respectively), alfalfa hay (CP, NDF, and ADF of 16.6%, 40.2%, and 30.2%, respectively) exhibits superior palatability, higher digestibility, and richer nutrition (Castells et al., 2012). In a limited number of studies on calves, providing alfalfa hay from the second week of age may be optimal, as feeding it too early (during the first week) could compromise nutrient digestibility and digestible nutrient intake (Xiao et al., 2023). Similarly, feeding alfalfa hay (or oat hay) too late (e.g., the 6th week of age) reduces DMI and growth performance (Hosseini et al., 2015; Lin et al., 2018). Nevertheless, determining the most appropriate age to introduce hay remains inconclusive, particularly for pre-weaning lambs. Furthermore, the establishment of rumen microflora may be the pivotal factor in rumen development among young ruminants. Yang et al. (2018) reported that, compared with lambs fed only starter pellets before 38 d of age, adding alfalfa hay at 10 d of age significantly increased the abundance of rumen fibrolytic bacteria, to improved feed intake and growth performance. Currently, there is a lack of research on the effects of adding alfalfa hay at 14 or 42 d of age on the rumen microbiota of lambs, as well as the sustained effects of such supplementation. The objectives of this study are to systematically evaluate the growth performance, plasma parameters, rumen epithelial microbiota composition, and rumen tissue gene expression of pre-weaning lambs in response to different timings of alfalfa hay supplementation. This study aims to provide a scientific reference for the feeding and management of lambs during the pre-weaning period.

## Material and Methods

### Animals, diets, and experimental design

All animal procedures were conducted according to the “Laboratory animal Guideline for ethical review of animal welfare” National Standard of the People’s Republic of China (GB/T 35892-2018). The care and use of animals fully complied with local animal welfare laws, guidelines, and policies.

**Figure 1.**
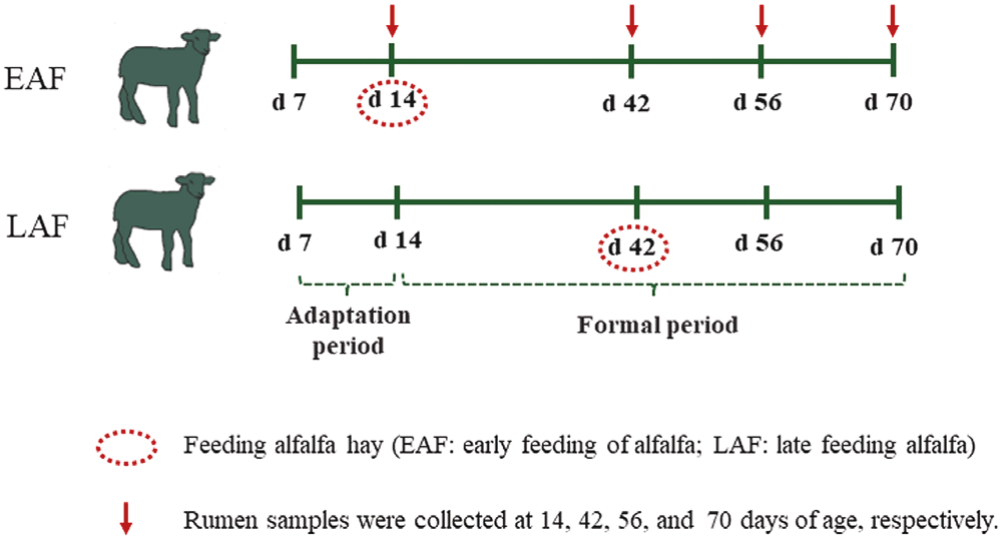
Experimental design and sampling schedule.

We selected 42 male 7-d-old Hu lambs (weighing 3.88 ± 0.92 kg) from parturient ewes (giving birth to 2 to 5 litters each) if an ewe gave birth to 2 male lambs, these 2 lambs were not assigned to the same treatment group. After a 7-d of adaptation period to their new environment and milk replacer (**MR**), the formal trial commenced and lasted for 8 wk. At the beginning of the experiment, 6 of 42 lambs were slaughtered at 14 d of age to serve as the control group (**CON**). The remaining 36 lambs were weighed, fed MR, starter pellets, and then randomly assigned to 2 feeding schemes based on their body weight (**BW**): the early alfalfa feeding group (**EAF**, *n* = 18), which received alfalfa hay supplementation starting at 14 d of age; and the late alfalfa feeding group (**LAF**, *n* = 18), which received alfalfa hay supplementation starting at 42 d of age (Figure 1). Daily intake of starter pellets and alfalfa hay was recorded based on the amount offered and refused by each pen from 14 to 70 d of age. The BW of each lamb was measured on a weekly basis before the morning feeding during the trial period. The DMI and ADG were calculated accordingly. Nutrient levels for MR, starter pellets, and alfalfa hay are shown in Table 1. Each unit, consisting of 3 lambs with similar BW, is housed in a separate pen. Two pens containing solid feed (for starter pellets and alfalfa hay) and one pen for water were placed inside the hutch. All lambs had free access to starter pellets, alfalfa hay, and water. To avoid wasting starter pellets and alfalfa hay during feeding, we adopted the principle of feeding less and adding more frequently. Each day, from 0800 to 2200 hours, starter pellets and alfalfa hay are added to the animals’ feed every 2 h, and the feeding amount is accurately recorded. The amount of starter pellets and alfalfa hay added is determined based on the amount of residual feed. The pens are cleaned and sterilized every 2 wk, in accordance with the standard operating procedures of the farm.

**Table 1.** Nutrient components of MR, starter pellets, and alfalfa hay (DM basis, %)

Items (%, unless otherwise stated)	Starter pellets	MR^1^	Alfalfa hay
Ingredients			
Corn	40.00	—	—
DDGS	3.00	—	—
Soybean meal	26.00	—	—
Wheat bran	13.00	—	—
Corn germ meal	6.00	—	—
Soybean hulls	4.00	—	—
Extruded soybean	4.00	—	—
Limestone	2.00	—	—
CaHPO_4_	0.50	—	—
NaCl	0.50	—	—
Premix^2^	1.00	—	—
Total	100.00	—	—
Chemical composition			
DM	89.70	94.45	86.63
CP	23.98	20.88	15.20
EE	4.86	11.08	2.23
NDF	22.46	—	55.96
ADF	8.10	—	43.07
Ash	7.48	2.59	7.09
Ca	0.98	1.05	1.35
P	0.72	0.68	0.16
Metabolic energy (MJ/Kg)^3^	11.67	14.16	7.96

DDGS, distillers dried grains with solubles; DM, dry matter; CP, crude protein; EE, ether extract; NDF, neutral detergent fibers; ADF, acid detergent fiber; Ash, crude ash.

^1^The MR was stored in powder, and was consisted with the whole milk powder, whey powder, protein concentrate, vitamin A (VA), VD_3_, VE, nicotinic acid, pantothenic acid, lysine, methionine, threonine, sodium chloride, copper, zinc manganese, and iron.

^2^Contained per kilogram of supplement: vitamin A, 800,000 IU; vitamin D_3_, 30,000 IU; vitamin E, 3,000 mg; Cu, 0.8 g; Fe, 4 g; Mn, 4 g; Zn, 5 g; I, 70 mg; Se, 20 mg; Co, 40 mg.

^3^Nutrient levels were all measured except the metabolic energy. The calculation method of metabolic energy refers to the method of Nutrient Requirements of Meat-type Sheep and Goat (NY/T 816-2021).

All lambs were artificially fed with MR from 7 to 70 d of age. To stimulate the consumption of starter pellets and alfalfa hay among lambs, 720 mL/d of MR (based on consumption during the adaptation period) was administered to the lambs at 15 d of age, and then this amount was gradually reduced by 40 mL/d until it reached 200 mL/d. All lambs were fed 4 times a day (at 0800, 1200, 1600, and 2000 hours) from 7 to 28 d of age, and 3 times a day (at 0800, 1200, and 1800 hours) from 29 to 70 d of age. The MR was dissolved in hot water, boiled, and then cooled to 45 to 50 °C, it was offered to the lambs when it was cooled to 38 ± 1 °C. The ratio of MR to water was 1:7 (with the weight of MR measured in grams and the volume of water measured in milliliters). Alfalfa hay was cut into 2.2 cm using a chaff cutter (Sida 9Z-4C; Luoyang Sida Agricultural Machinery Co., Ltd, Luoyang, China).

The fecal score was recorded daily on an individual basis from 14 to 28 d of age (no diarrhea was observed after 28 d of age) using the methods described by Larson et al. (1977) (Table 2). If an animal presented a fecal score ≥ 4 for 2 consecutive days or a fecal score ≥ 3 for 3 consecutive days, it was considered diarrhea. The frequency of diarrhea was calculated as [(the number of lambs with diarrhea × days of diarrhea) / (the total number of lambs × the number of experimental days)] × 100%.

**Table 2.** Feces scoring criteria

Shape	Fluidity	Score
Normal	Firm but not hard	1
Soft	Does not hold form, piles but spreads slightly (i.e., soft serve ice milk)	2
Runny	Spreads readily to about 2 to 3 mm depth (i.e., pancake batter)	3
Watery	Liquid consistency, splatters (i.e., orange juice)	4

### Feed sampling and total-tract apparent digestibility analysis

Fresh fecal samples (approximately 30 g each) were collected from each lamb twice daily through rectal stimulation near the end of the last 7 d of the feeding trial. The fecal samples were pooled by pen and subsequently frozen at −20 °C for later analysis. Meanwhile, representative samples of MR, starter pellets, and alfalfa hay were also collected twice daily during the last 7 d of the feeding trial. Total-tract apparent digestibility was determined using acid-insoluble ash (**AIA**) as an internal marker, as described by Ponce et al. (2013). The feed and fecal samples were dried at 60 °C for 72 h and then ground in a roller mill to pass through a 1.0-mm sieve. Subsequently, their chemical compositions, including dry matter (**DM**, AOAC method 930.15), CP (AOAC method 984.13), ether extract (**EE**, AOAC method 920.39), and crude ash (**Ash**, AOAC method 924.05), were analyzed according to the methods published by the Association of Official Analytical Chemists (AOAC) in 2007. NDF and ADF were analyzed using the ANKOM fiber analysis equipment (A2000i; Ankom Technology Corp., Fairport, NY, USA), as described by Van Soest et al. (1991). The equation used to calculate digestibility was 100% − [(nutrient in feces, % × AIA in feed, %) / (nutrient in feeds, % × AIA in feces, %)] × 100%.

### Rumen and blood samples

Six lambs per treatment (one lamb from each pen) were randomly sacrificed 3 h after the morning feeding, at the ages of 42, 56, and 70 d. The abdominal cavity was dissected, and the reticulorumen, omasum, abomasum, and intestines were separated. The contents of these organs were then poured out and weighed. Immediately, 2 pieces of rumen wall tissue (each 0.5 × 0.5 cm) were collected from the ventral sac and washed 3 times with ice-cold phosphate buffer saline (**PBS**) until the PBS became clear. One of the rumen wall tissue pieces was used for RNA extraction. Another piece of rumen wall tissue was separated from the muscular and serosal layers through blunt dissection, in order to collect the rumen epithelium for epithelium-associated microbiota analysis. These samples were immediately frozen in liquid nitrogen and then stored at −80 °C.

Approximately 20 mL of blood was collected from the jugular vein prior to slaughter. Of this, 10 mL of blood was placed in a non-anticoagulant tube for serum preparation, while another 10 mL was placed in a heparinized tube for plasma preparation. Subsequently, the blood samples were allowed to coagulate for 20 min and were then centrifuged at 3,500 × *g* for 15 min at 4 °C. The samples were stored at −20 °C until further analysis. Using sheep ELISA kits from Wuhan Jiyinmei Biotechnology (Wuhan, China), the serum concentrations of lipopolysaccharide (**LPS**), immunoglobulins (IgG, IgM, and IgA), interleukins (IL-1β, IL-2, IL-4, and IL-6), and tumor necrosis factor-α (**TNF-α**) were determined, all in accordance with the manufacturer’s instructions. Using commercial ELISA kits from Beijing Solarbio Science & Technology (Beijing, China), the serum concentration of β-hydroxybutyric acid (**BHBA**) was determined, following the manufacturer’s instructions. Similarly, the concentrations of alkaline phosphatase (**ALP**), albumin (**ALB**), triglyceride (**TG**), high-density lipoprotein cholesterol (**HDL-C**), total protein (**TP**), urea (**UREA**), creatinine (**CRE**), and glucose (**GLU**) in plasma were measured using an automatic biochemical analyzer (Hitachi 3110, Hitachi High-Technologies Corporation, Tokyo, Japan), with corresponding commercial test kits provided by Lepu Diagnostic Technology Co., Ltd (Beijing, China), also according to the manufacturer’s instructions.

### The rumen tissue RNA extraction, reverse transcription, and quantitative real-time polymerase chain reaction

Total RNA was extracted from rumen wall tissue using the TRIzol reagent (TaKaRa Biotechnology Co. Ltd, Dalian, China) according to the manufacturer’s instructions. Then, the purity and quantity of the total RNA were evaluated using an ultraviolet spectrophotometer (Pultton P200CM, San Jose, CA, USA) and adjusted to a uniform concentration of 500 ng/μL with ddH_2_O. Two microliters of total RNA (500 ng/μL) from each sample were reverse transcribed to cDNA using the *Evo M-MLV* RT Mix Kit with gDNA Clean for qPCR Ver.2 (Accurate Biotechnology Co., Ltd, Hunan, China) on a LifeECO thermal cycler (TC-96/G/H(b)C, BIOER, Hangzhou, China). The reactions were incubated at 37 °C for 15 min, followed by 85 °C for 5 s.

Quantitative real-time polymerase chain reaction (**qRT-PCR**) was performed using the LightCycler 480 II real-time PCR system (Roche Diagnostics, Indianapolis, IN, USA) with a SYBR Green Premix *Pro Taq* HS qPCR Kit (Rox Plus) (Accurate Biotechnology Co., Ltd, Hunan, China). Fluorescence quantitative PCR was performed to detect the mRNA expressions of *β-actin*, *Claudin-1*, *Claudin-4*, *Occludin*, *zonula occludens-1* (***ZO-1***), *IL-1β*, *IL-10*, *tumor necrosis factor-α* (***TNF-α***), *insulin-like growth factor 1* (***IGF-1***), and *transforming growth factor-β* (***TGF-β***). The qRT-PCR primers and amplification conditions for this study are listed in Table 3. Each sample was run in a 10-μL reaction mixture, including 5 μL 2 × SYBR Green PCR Master Mix, 0.2 μL of each primer (0.2 μM), 3.6 μL ddH_2_O and 1 μL DNA templates. The relative mRNA expression levels were normalized to the expression of *β-actin* (a normal housekeeping gene), and the data were calculated using the 2^−ΔΔCT^ method.

**Table 3.** The primer sequencings designed and amplification conditions for qRT-PCR

Genes^1^	Forward primer sequence (5ʹ to 3ʹ)	Reverse primer sequence (5ʹto 3ʹ)	Tm (°C)
*β-Actin*	TCCGTGACATCAAGGAGAAGC	CCGTGTTGGCGTAGAGGT	55
*IGF-1*	GCTCTCAACATCTCCCATCTCC	CCCATTGCTTCTGAAGTGCAAA	60
*Claudin-1*	GTGGATGTCGTGCGTGTC	TAGTCCCAGCAGGATGCC	58
*Occludin*	AGCAGCAGTGGTAACTTGG	TCCCGTCGTGTAGTCTGTT	58
*ZO-1*	CGAGCAGACGCAGAAAA	GGCAGAAGATTGTGGTTGA	55
*IL-1β*	CAGCCGTGCAGTCAGTAA	TGTGAGAGGAGGTGGAGAG	57
*IL-10*	GGCGCTGTCATCGTTTTCTG	ACACCCCTCTCTTGGAGCAT	60
*TNF-α*	ACACCATGAGCACCAAAAGC	AGGCACCAGCAACTTCTGGA	60
*Claudin-4*	ACTGCGTGGATGATGAGAGC	GAAGTCACGGATGACGTTGTT	61
*TGF-β*	TGACCCACAGAGAGGAAATAGA	AACCCGTTGATGTCCACTTGAA	55

^1^IGF-1, insulin-like growth factor 1; ZO-1, zonula occludens-1; IL-1β, interleukin-1β; IL-10, interleukin-10; TNF-α, tumor necrosis factor-α; TGF-β, transforming growth factor-β.

### The rumen epithelium microbial DNA extraction, PCR amplification, 16S rRNA sequencing, and data processing

After the samples were thawed, the DNA from the rumen epithelium microbiota was extracted, following the manufacturer’s instructions for the commercial DNA Kit (Omega Bio-tek, Norcross, GA, U.S.), and the integrity of the DNA was analyzed using 1% agarose gel electrophoresis. Its purity was evaluated by a NanoDrop 2000 UV spectrophotometer (ThermoFisher, Waltham, MA, USA) based on the 260:280 nm ratio (>1.8). All the DNA samples were stored at −80 °C until used for sequencing analysis.

Using the universal primers 338F-806R, in conjunction with primers containing specific barcodes for each sample, we amplified the V3 and V4 regions of the bacterial ribosomal RNA genes, Following PCR amplification, all amplicon libraries were then sequenced utilizing the Illumina MiSeq platform.

The raw FASTQ files were demultiplexed and quality-filtered using default parameters in QIIME 2 (version 1.9.1) (Caporaso et al., 2010), with the following criteria: 1) reads of 300 base pairs were truncated at any site where the average quality score was less than 20 over a sliding window of 50 base pairs, and those truncated to less than 50 base pairs were discarded; 2) paired-end reads were merged into a single sequence if they had an overlap of at least 10 base pairs; 3) the maximum allowed mismatch ratio in the overlap region of the merged sequence was 0.2, and sequences failing to meet this criterion were screened out; 4) the samples were differentiated based on the barcode and primers present at both ends of the sequence, with the sequence direction adjusted accordingly. For the barcode, no mismatches were allowed, and the maximum number of mismatches allowed for the primers was 2.

Paired-end reads were merged using FLASH software (version 1.2.11) based on overlapping regions (Magoc and Salzberg, 2011). Operational taxonomic units (**OTUs**) were clustered using UPARSE (version 11) at a 97% identity threshold (Edgar, 2013). The most abundant sequences in OTUs were selected as representative sequences and aligned against the bacterial database of SILVA (version 138) using the RDP Classifier (version 11.5) Bayesian algorithm. Alpha diversities (Sobs, Shannon, and Chao index) and beta diversities were calculated in QIIME software. The principal co-ordinates analysis (**PCoA**) plot of beta diversity was visualized using the ‘ggplot2’ package in R (v3.3.1) (Wang et al., 2012). Analysis of similarity (**ANOSIM**) was performed to examine significant differences in beta diversity. Wilcoxon rank-sum test analysis was used to analyze the effect of treatment on the composition of the rumen epithelial microbiota. Linear discriminant analysis Effect Size (**LEfSe**) analysis was conducted to investigate the impact of age on the composition of the rumen epithelial microbiota.

### Statistical analysis

The data on growth performance, organ weight, and blood variables of lambs were analyzed using a 2-way ANOVA through the PROC GLM procedure in SAS 9.2, aiming to investigate the response to treatment, age, and their interaction. The initial BW was incorporated as a covariate (covariate structure: compound symmetry) in the statistical analysis of ADG. The statistical model is as follows:


$$\begin{array}{l} Y_{ij}=\mu +T_{i}+P_{j}+T{P_{i}}_{j}+\varepsilon_{ij} \end{array}$$

where *Y*_*ij*_ is the dependent variable, μ is the overall mean, *T*_*i*_ is the treatment effect, *P*_*j*_ is the age effect, *TP*_*ij*_ is the interaction of treatment and age, and *ε*_*ij*_ is the error term.

Data on total-tract apparent digestibility and diarrhea frequency were analyzed according to a one-way ANOVA. The statistical model is as follows:


$$\begin{array}{l} Y_{i}=\mu +T_{i}+\varepsilon_{i} \end{array}$$

Where *Y*_*i*_ is the dependent variable, μ is the overall mean, *T*_*i*_ is the treatment effect, and ε_*i*_ is the error term.

Multiple comparisons of means among treatments were performed using Tukey’s multiple-range tests. For all the analyses, a *P* ≤ 0.05 was considered to indicate a significant difference. All statistical analyses were performed using SAS 9.2 (SAS Inst. Inc., Cary, NC, USA).

## Results

### Growth performance, feed intake, and diarrhea frequency

**Figure 2.**
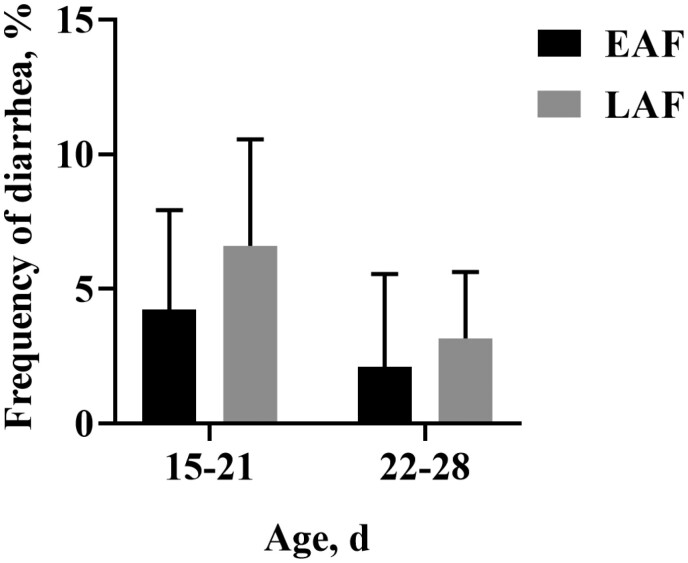
Diarrhea frequency of lambs in the EAF and LAF groups. Lambs were fed alfalfa hay at 14 d of age as early alfalfa hay feeding group (EAF) or lambs were fed alfalfa hay at 42 d of age as late alfalfa hay feeding group (LAF).

Animal growth performance and feed intake are presented in Table 4. No significant differences were observed among the EAF and LAF groups regarding initial BW (*P* > 0.05) and final BW (*P* > 0.05). From 14 to 42 d of age, ADG (*P* < 0.01), starter intake (*P* < 0.01), and TDMI (*P* < 0.01) increased with age, and ADG (*P* < 0.01), starter intake (*P* = 0.01), and TDMI (*P* < 0.01) were greater in the EAF group than the LAF group. From 43 to 70 d of age, starter intake (*P* < 0.01), alfalfa hay intake (*P* < 0.01), TDMI (*P* < 0.01), and feed efficiency (**FE**) (*P* = 0.03) increased with age. However, there were no significant differences between the EAF and LAF groups in starter intake (*P* = 0.52), alfalfa hay intake (*P* = 0.93), TDMI (*P* = 0.54), and FE (*P* = 0.19). During the entire trial period, age had a significant impact on ADG (*P* < 0.01), starter intake (*P* < 0.01), and TDMI (*P* < 0.01). Additionally, from 43 to 70 d of age, treatment and age interactively affected alfalfa hay intake (*P* = 0.02). Regarding diarrhea frequency, no statistical difference was found between the EAF and LAF groups (Figure 2). However, we observed that in the first week of the trial (15 to 21 d of age), the diarrhea frequency was 4.23% in EAF lambs and 6.61% in LAF lambs. Similarly, in the second week of the experiment (22 to 28 d of age), the diarrhea frequency was 2.12% in the EAF group and 3.17% in the LAF group. This suggests that early supplementation of alfalfa hay may reduce diarrhea frequency in lamb before weaning, as diarrhea was predominantly observed in lambs under 28 d of age. Therefore, we focused our analysis on the first 2 wk of the trial.

**Table 4.** Effect of alfalfa hay supplementation time on performance and feed intake of lambs

Item	Trt		SEM	*P* value		
	EAF	LAF		Trt	Age1	Trt × Age
Initial BW, kg	3.72	3.69	0.06	0.85	—	—
Final BW, kg	14.19	14.05	0.69	0.93	—	—
ADG, kg/d						
Entire trial	0.21	0.19	0.01	0.39	<0.01	0.41
14 to 42 d of age	0.14	0.10	0.01	<0.01	<0.01	0.26
43 to 70 d of age	0.27	0.29	0.01	0.52	0.30	0.74
Starter intake, g of DM/d						
Entire trial	274.56	251.99	19.32	0.12	<0.01	0.99
14 to 42 d of age	124.08	97.64	11.54	0.01	<0.01	0.44
43 to 70 d of age	425.04	406.34	19.68	0.52	<0.01	0.97
Alfalfa hay intake, g of DM/d						
Entire trial	34.17	—	—	—	—	—
14 to 42 d of age	12.88	—	—	—	—	—
43 to 70 d of age	55.46	55.83	2.67	0.93	<0.01	0.02
TDMI, g of DM/d						
Entire trial	349.07	319.82	20.83	0.07	<0.01	0.93
14 to 42 d of age	186.48	146.31	10.24	<0.01	<0.01	0.13
43 to 70 d of age	511.66	493.33	21.14	0.54	<0.01	0.95
FE						
Entire trial	1.72	1.88	0.11	0.32	0.22	0.12
14 to 42 d of age	1.47	2.05	0.20	0.12	0.29	0.21
43 to 70 d of age	2.03	1.69	0.09	0.19	0.03	0.34

EAF, lambs were fed alfalfa hay at 14 d of age as early alfalfa feeding group; LAF, lambs were fed alfalfa hay at 42 d of age as late alfalfa feeding group; BW, body weight; ADG, average daily gain; DM, dry matter; TDMI, total dry matter intake (MR, starter feed, and alfalfa hay); SEM, standard error mean; Trt, treatment effect; FE, feed efficiency, defined as TDMI/ADG.

^1^For all variables, data were summarized by week.

### Total-tract apparent digestibility

There was no significant difference (*P* > 0.05) in total-tract apparent digestibility of DM, CP, EE, NDF, and Ash between treatments (Table 5). However, we found that the digestibility of NDF and ADF in the lambs of the EAF group increased by 5.08% and 5.95%, respectively, compared to those in the LAF group.

**Table 5.** Effect of alfalfa hay supplementation time on the apparent digestibility of nutrients in lambs

Items, %	EAF	LAF	SEM	*P* value
DM	77.50	78.69	2.30	0.81
CP	79.27	81.05	2.24	0.71
EE	85.12	84.98	1.85	0.97
NDF	50.59	45.51	4.08	0.51
ADF	48.49	42.54	2.08	0.32
Ash	55.52	56.19	3.84	0.94

EAF, lambs were fed alfalfa hay at 14 d of age as early alfalfa feeding group; LAF, lambs were fed alfalfa hay at 42 d of age as late alfalfa feeding group; SEM, standard error of the mean; DM, dry matter; CP, crude protein; EE, ether extract; NDF, neutral detergent fibers; ADF, acid detergent fiber; Ash, crude ash; SEM, standard error mean.

### Anatomical development of the gastrointestinal tract

For both groups, the weight of reticulorumen (*P* < 0.01), omasum (*P* < 0.01), abomasum (*P* < 0.01), whole stomach (*P* < 0.01), intestine (*P* < 0.01), and gastrointestinal (**GIT**) (*P* < 0.01) significantly increased with age. Additionally, the intestinal weight was significantly affected by the treatment (*P* = 0.04) (Table 6). Compared with the LAF group, the intestinal weight of lambs in the EAF group increased by 30.57%, 10.68%, and 9.08% at 42, 56, and 70 d of age, respectively. Similarly, the reticulorumen weight of lambs in the EAF group increased by 21.28%, 15.03%, and 7.09% at 42, 56, and 70 d of age, respectively, suggesting that early feeding of alfalfa hay can increase the weight of both reticulorumen and intestine, but the effect decreases with age. The reticulorumen weight relative to whole stomach weight increased from 14 to 56 d of age for both groups (*P* = 0.05). In contrast, the abomasum weight relative to the whole stomach weight decreased over the same period (*P* < 0.01). After 56 d of age, the ratios of reticulorumen weight to whole stomach and abomasum weight to whole stomach gradually stabilized. Both treatment and age interactively affected the omasum weight relative to whole stomach weight (*P* = 0.04). From 14 to 56 d of age, the ratios of reticulorumen weight (*P* < 0.01), omasum weight (*P* < 0.01), and GIT weight (*P* < 0.01) relative to BW increased for both groups. After 56 d of age, these ratios gradually stabilized. The ratios of intestine weight (*P* = 0.02) and GIT weight (*P* = 0.02) relative to BW were affected by the treatment.

**Table 6.** Effect of age and alfalfa supplementation time on anatomical development of gastrointestinal tract of lambs

Items	Trt	Day of age				SEM	*P* value		
		14	42	56	70		Trt	Age	Trt * Age
Reticulorumen, g	EAF	21.33^d^	164.85^c^	319.18^ab^	366.96^a^	17.44	0.08	<0.01	0.90
	LAF		129.77^c^	271.22^b^	340.93^a^				
Omasum, g	EAF	2.58^d^	11.18^c^	22.83^b^	28.98^a^	1.69	0.26	<0.01	0.32
	LAF		6.98^cd^	20.20^b^	30.47^a^				
Abomasum, g	EAF	26.13^e^	55.68^cd^	82.58^ab^	98.23^a^	4.51	0.11	<0.01	0.88
	LAF		43.03^de^	72.15^bc^	92.60^a^				
Whole stomach, g	EAF	50.05^d^	225.50^c^	415.12^ab^	470.72^a^	25.58	0.23	<0.01	0.78
	LAF		179.78^c^	370.62^b^	464.00^a^				
Intestine, g	EAF	187.91^d^	366.92^c^	576.65^ab^	705.20^a^	32.32	0.04	<0.01	0.86
	LAF		254.74^cd^	515.05^b^	641.15^a^				
GIT, g	EAF	237.96^d^	586.25^c^	991.77^ab^	1175.92^a^	57.36	0.08	<0.01	0.87
	LAF		434.53^cd^	873.40^b^	1105.15^a^				
Relative to whole stomach weight, %									
Reticulorumen	EAF	42.74^c^	69.09^b^	74.31^a^	72.34^ab^	1.82	0.15	0.05	0.6
	LAF		72.37^ab^	74.92^a^	73.50^ab^				
Omasum	EAF	5.15^ab^	6.35^a^	5.59^a^	6.23^a^	0.24	0.12	0.09	0.04
	LAF		3.86^b^	5.47^ab^	6.58^a^				
Abomasum	EAF	52.12^a^	31.70^b^	19.72^c^	21.20^c^	1.93	0.13	<0.01	0.25
	LAF		23.77^c^	19.53^c^	19.91^c^				
Relative to BW before slaughter, %									
Reticulorumen	EAF	0.57^e^	2.17^cd^	3.02^a^	2.56^b^	0.13	0.11	<0.01	0.85
	LAF		2.03^d^	2.75^ab^	2.42^bc^				
Omasum	EAF	0.07^c^	0.15^b^	0.22^a^	0.22^a^	0.01	0.15	<0.01	0.44
	LAF		0.11^bc^	0.20^a^	0.22^a^				
Abomasum	EAF	0.70	0.74	0.78	0.74	0.02	0.13	0.69	0.98
	LAF		0.68	0.71	0.66				
Intestine	EAF	5.08^ab^	4.92^ab^	5.51^a^	5.22^a^	0.14	0.02	0.09	0.86
	LAF		4.03^b^	4.99^ab^	4.55^ab^				
GIT	EAF	6.42^d^	7.80^bc^	9.43^a^	8.72^ab^	0.22	0.02	<0.01	0.98
	LAF		6.85^cd^	8.64^ab^	7.84^bc^				

EAF, lambs were fed alfalfa hay at 14 d of age as early alfalfa feeding group; LAF, lambs were fed alfalfa hay at 42 d of age as late alfalfa feeding group; gastrointestinal tract (GIT); SEM, standard error mean. Trt, treatment effect.

### Serum parameters

The effect of the timing of alfalfa hay supplementation on the blood biochemical indices of pre-weaning lambs is shown in Table 7. In terms of the treatment effect, no significant difference was observed in the concentration of ALP (*P* = 0.28), ALB (*P* = 0.30), TG (*P* = 0.17), HDL-C (*P* = 0.26), TP (*P* = 0.85), UREA (*P* = 0.62), CRE (*P* = 0.48), and GLU (*P* = 0.72) between the 2 treatments. Regarding the age effect, in the EAF groups, the concentration of CRE was greater at 42 and 56 d compared to 70 d of age (*P* = 0.04). Across all groups, the concentration of GLU was greater at 14 d than at 42, 56, or 70 d of age (*P* < 0.01). Additionally, the concentrations of ALP (*P* = 0.03), ALB (*P* < 0.01), TP (*P* = 0.03), and UREA (*P* = 0.04) increased over time in both groups. However, no significant interactive effect of treatment and age was observed on any of the serum parameters (*P* > 0.05).

**Table 7.** Effect of alfalfa hay supplementation time on blood biochemistry indices of pre-weaning lambs

Items	Trt	Day of age				SEM	*P* value		
		14	42	56	70		Trt	Age	Trt* Age
ALP, U/L	EAF	482.28^bc^	438.39^c^	459.11^c^	732.99^a^	29.07	0.28	0.03	0.12
	LAF		509.83^abc^	699.97^ab^	635.55^abc^				
ALB, g/L	EAF	28.16^d^	29.49^cd^	32.65^abc^	35.39^a^	0.51	0.30	<0.01	0.42
	LAF		29.61^bcd^	32.31^abc^	32.78^ab^				
TG, mmol/L	EAF	0.30	0.27	0.37	0.35	0.02	0.17	0.52	0.76
	LAF		0.38	0.39	0.41				
HDL-C, mmol/L	EAF	12.04	5.10	5.04	5.39	0.39	0.26	0.62	0.84
	LAF		5.59	5.28	5.53				
TP, g/L	EAF	52.33^ab^	49.58^b^	51.78^ab^	58.02^a^	0.84	0.85	0.03	0.32
	LAF		51.31^ab^	53.00^ab^	54.13^ab^				
UREA, mmol/L	EAF	4.59^c^	7.26^ab^	8.67^ab^	8.20^ab^	0.31	0.62	0.04	0.29
	LAF		6.71^b^	7.61^ab^	9.05^a^				
CRE, μmol/L	EAF	40.04^ab^	44.77^a^	45.19^a^	33.70^b^	1.19	0.48	0.04	0.19
	LAF		37.20^ab^	43.50^a^	37.99^ab^				
GLU, mmol/L	EAF	6.67^a^	4.34^cd^	5.26^bc^	5.52^b^	0.18	0.72	<0.01	0.76
	LAF		4.02^d^	5.44^b^	5.35^bc^				

EAF, lambs were fed alfalfa hay at 14 d of age as early alfalfa feeding group; LAF, lambs were fed alfalfa hay at 42 d of age as late alfalfa feeding group; Trt, treatment effect; SEM, standard error mean.

The effect of the timing of alfalfa hay supplementation on the concentrations of serum immune indices, LPS, and BHBA in pre-weaning lambs is shown in Table 8. For the treatment effect, the concentrations of serum IgA (*P* < 0.01), IgG (*P* = 0.02), IL-2 (*P* < 0.01), and IL-6 (*P* < 0.01) were significantly affected by the treatment. Specifically, at 42 d of age, the serum IgA concentration was greater in the EAF group compared to the LAF group (*P* < 0.01). For the age effect, both groups showed an increase in serum IgA (*P* < 0.01) and IgG (*P* = 0.03) concentrations from 42 to 70 d of age. Additionally, the concentrations of serum IL-1β (*P* < 0.01), IL-4 (*P* < 0.01), TNF-α (*P* < 0.01), LPS (*P* < 0.01), and BHBA (*P* = 0.02) increased from 14 to 70 d of age. Notably, the concentration of IL-1β (*P* < 0.01) was greater at 70 than at 14, 42, and 56 d of age for both groups. In the EAF group, the concentration of IL-4 was greater at 70 than at 14 d of age (*P* < 0.01), while TNF-α was greater at 56 and 70 than at 14 and 42 d of age (*P* < 0.01). However, in the LAF group, the concentration of IL-4 (*P* < 0.01) and TNF-α (*P* < 0.01) were greater at 70 d than at 14, 42, and 56 d of age. Furthermore, serum IL-2 (*P* < 0.01) and IL-6 (*P* < 0.01) concentrations increased from 14 to 70 d of age in both groups. Specifically, the EAF lambs had greater concentrations of IL-2 (*P* < 0.01) and IL-6 (*P* < 0.01) at 70 than at 14 and 42 d of age, and the LAF lambs exhibited a greater concentration of IL-6 (*P* < 0.01) at 70 d than at 14 d of age.

**Table 8.** Effect of alfalfa hay supplementation time on the concentration of serum immune, LPS and BHBA indices of pre-weaning lambs

Items	Trt	Day of age				SEM	*P* value		
		14	42	56	70		Trt	Age	Trt* Age
IgA, μg/mL	EAF	10.07^ab^	9.73^b^	10.34^ab^	10.89^a^	0.15	<0.01	<0.01	0.70
	LAF		8.53^c^	9.59^b^	10.13^ab^				
IgG, μg/mL	EAF	25.21^ab^	24.82^ab^	26.25^a^	27.12^a^	0.42	0.02	0.03	0.79
	LAF		21.95^b^	23.81^ab^	25.77^a^				
IgM, μg/mL	EAF	24.61	25.29	25.23	25.98	0.39	0.29	0.12	0.46
	LAF		24.06	23.44	26.49				
IL-1β, pg/mL	EAF	234.78^b^	246.95^b^	248.15^b^	298.22^a^	5.55	0.37	<0.01	0.61
	LAF		227.86^b^	252.10^b^	287.38^a^				
IL-2, pg/mL	EAF	212.09^bc^	224.36^bc^	235.77^ab^	250.71^a^	3.38	0.02	<0.01	0.62
	LAF		207.42^c^	229.37^abc^	230.03^abc^				
IL-4, pg/mL	EAF	114.36^c^	123.23^bc^	125.58^abc^	135.12^ab^	2.18	0.84	<0.01	0.58
	LAF		119.35^bc^	121.55^bc^	140.44^a^				
IL-6, pg/mL	EAF	128.30^d^	131.71^cd^	140.15^bcd^	150.90^ab^	1.83	0.04	<0.01	0.59
	LAF		145.06^abc^	145.35^abc^	156.20^a^				
TNF-α, pg/mL	EAF	213.55^c^	212.03^c^	247.85^ab^	267.17^a^	5.01	0.18	<0.01	0.49
	LAF		210.66^c^	221.77^bc^	259.08^a^				
LPS, ng/mL	EAF	110.22^b^	116.02^b^	110.97^b^	136.52^a^	1.93	0.19	<0.01	0.44
	LAF		117.47^b^	120.51^b^	137.47^a^				
BHBA, μmol/mL	EAF	0.54^c^	0.81^b^	0.82^ab^	0.85^ab^	0.02	0.47	0.02	0.31
	LAF		0.77^b^	0.85^ab^	0.96^a^				

EAF, lambs were fed alfalfa hay at 14 d of age as early alfalfa feeding group; LAF, lambs were fed alfalfa hay at 42 d of age as late alfalfa feeding group; SEM, standard error mean; Trt, treatment effect.

### Effect of hay supplementation timing on relative mRNA expression in the rumen wall in pre-weaning lambs

**Figure 3.**
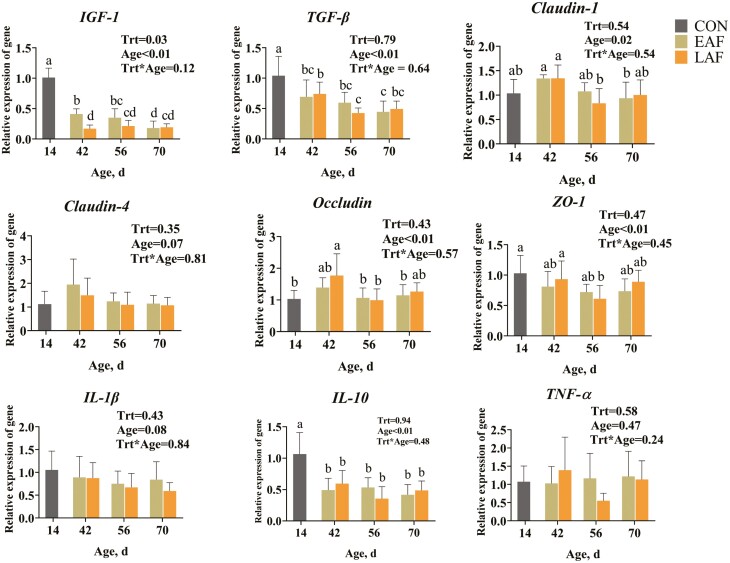
Relative mRNA expression of rumen wall proliferation, tight junction proteins, and cytokines in lambs of EAF and LAF groups at different ages. Trt, treatment effect. EAF, lambs were fed alfalfa hay at 14 d of age as early alfalfa feeding group; LAF, lambs were fed alfalfa hay at 42 d of age as late alfalfa feeding group; CON, lambs were slaughtered at 14 d of age.

The mRNA expression of *IGF-1*, *TGF-β*, *Claudin-1*, *Claudin-4*, *Occludin*, *ZO-1*, *IL-1β*, *IL-10*, and *TNF-α* in the rumen wall was determined (Figure 3). No significant differences were observed between the EAF and LAF groups in terms of the relative mRNA expression of *TGF-β* (*P* = 0.79), *Claudin-1* (*P* = 0.54), *Claudin-4* (*P* = 0.35), *Occludin* (*P* = 0.43), *ZO-1* (*P* = 0.47), *IL-1β* (*P* = 0.43), *IL-10* (*P* = 0.94), and *TNF-α* (*P* = 0.58). The relative expression of *IGF-1*, *TGF-β*, and *IL-10* in the rumen wall was significantly greater at 14 d than at 42, 56, and 70 d of age (*P* < 0.01). Among them, *IGF-1* mRNA expression was significantly greater in the EAF group than in the LAF group at 42 d of age (*P* < 0.01). Furthermore, the gene expression of *TGF-β* (*P* < 0.01), *Claudin-1* (*P* = 0.02), *Occludin* (*P* < 0.01), and *ZO-1* (*P* < 0.01) were greater at 42 d of age than at 56 d of age in the LAF group. However, no difference was observed in the EAF group between 42 and 56 d of age for these genes.

### Diversity of the bacterial community

In this study, 16S rRNA gene sequence analysis of rumen epithelial bacterial samples generated a total of 2,268,472 high-quality sequences, with a mean of 55,327 ± 6,848 sequences per sample. Based on a 97% similarity threshold, the overall number of OTUs detected was 14,961, averaging 356 ± 89 OTUs per sample. The coverage exceeded 0.99, indicating that our data provide sufficient sequencing depth to comprehensively represent the rumen epithelial bacterial composition across all tested animals.

**Figure 4.**
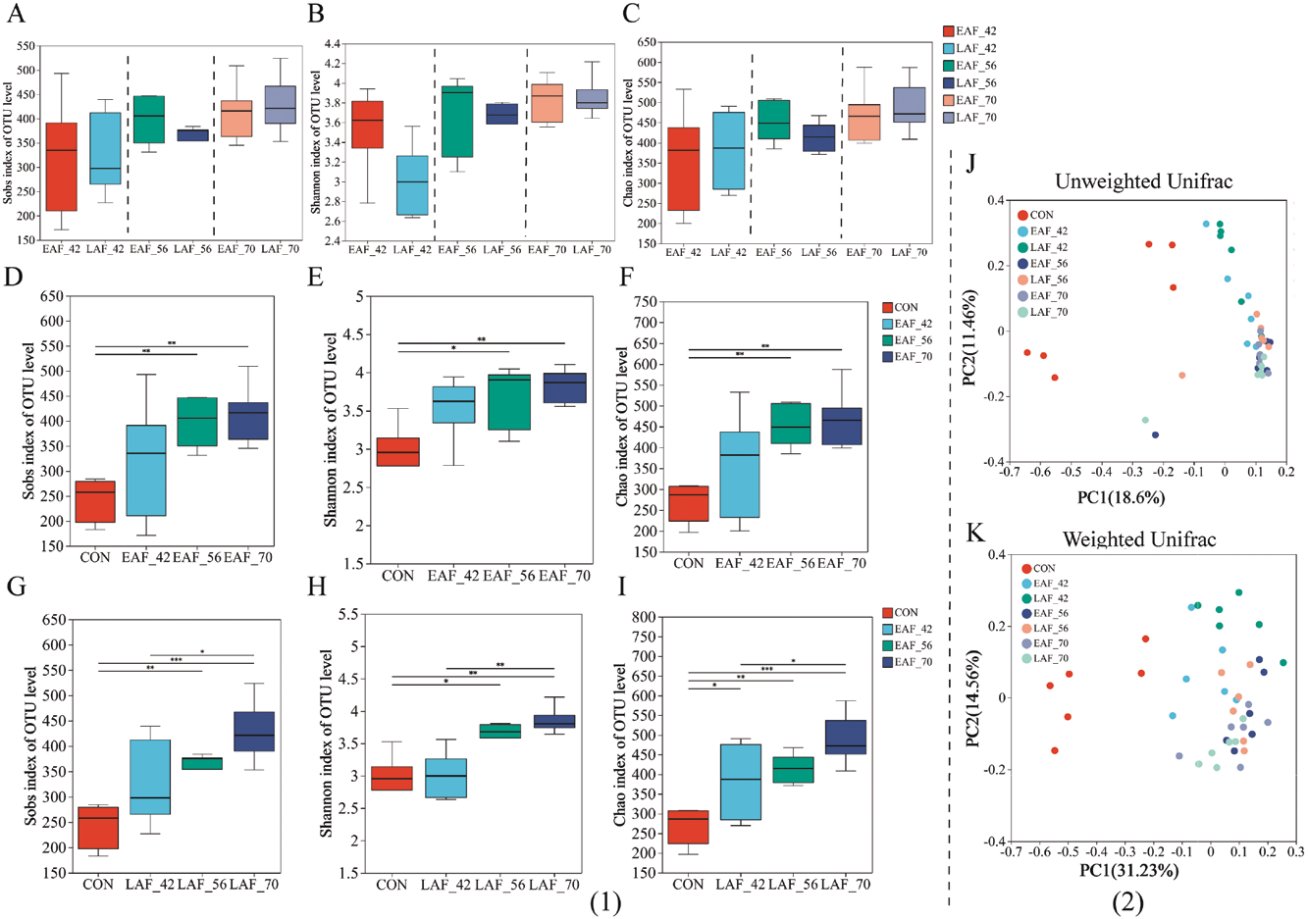
The effect of hay supplementation timing on alpha and beta diversity of rumen epithelial bacteria in pre-weaning lambs. (1) Sobs (A), Shannon (B), and Chao (C) indices in the rumen epithelial bacteria between the EAF and LAF groups at 42, 56, and 70 d of age. Alpha diversity of the rumen epithelial bacteria was analyzed using the Kruskal–Wallis test and a post-hoc Tukey–Kramer multiple comparison, and the FDR method was used for *P* value correction. The rumen bacterial alpha diversities of EAF group at 14, 42, 56, and 70 d of age (D, E, F), and the alpha diversities of LAF group at 14, 42, 56, and 70 d of age (G, H, I). * *P* < 0.05, ** *P* < 0.01, *** *P* < 0.001. (2) The principal coordinate analysis (PCoA) was performed based on unweighted (J) and weighted (K) Unifrac distances. EAF, lambs were fed alfalfa hay at 14 d of age as early alfalfa feeding group; LAF, lambs were fed alfalfa hay at 42 d of age as late alfalfa feeding group; CON, lambs were slaughtered at 14 d of age.

There were no significant differences in the Sobs (Figure 4A), Shannon (Figure 4B), and Chao (Figure 4C) indices between the EAF and LAF groups at 42, 56, and 70 d of age, respectively (*P* > 0.05). Subsequently, changes in alpha diversity were analyzed for the EAF and LAF groups at the ages of 42, 56, and 70 d. The Sobs, Shannon, and Chao indices increased with age in both the EAF and LAF groups. Notably, at 56 and 70 d of age, the Sobs (*P* < 0.01) (Figure 4D), Shannon (*P* < 0.05) (Figure 4E), and Chao (*P* < 0.01) (Figure 4F) indices were greater in the EAF group compared to the CON group. In the LAF group, the Sobs (*P* < 0.01) (Figure 4G) and Shannon (*P* < 0.01) (Figure 4H) indices were greater at 56 and 70 d of age than at 14 d of age (CON group). Additionally, the Sobs and Shannon indices at 70 d of age were significantly higher than those at 42 d of age (*P* < 0.05). Similarly, the Chao index (*P* < 0.05) (Figure 4I) in the LAF group was greater at 42, 56, and 70 d of age than at 14 d of age (CON group), and it was also greater at 70 d of age compared to 42 d of age (*P* < 0.05).

The beta-diversity analysis showed that the bacterial community’s unweighted (Figure 4J) and weighted (Figure 4K) Unifrac distances were significantly influenced by age (Unweighted Unifrac ANOSIM, *R* = 0.37, *P* = 0.001; Weighted Unifrac ANOSIM, *R* = 0.36, *P* = 0.001). Notably, this age-related effect was particularly pronounced at 14 and 42 d of age, with distinct clustering patterns observed at these time points (Figure 4J and K).

### The composition of the bacterial phyla, and genera

**Figure 5.**
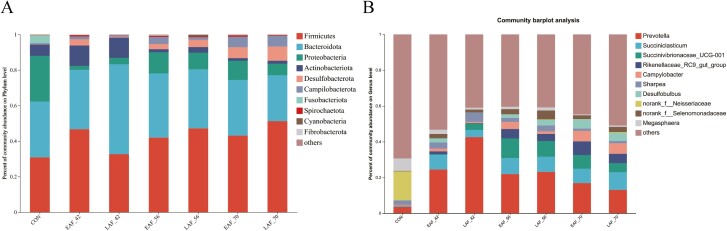
The composition of the rumen epithelial bacteria in response to hay supplementation timing. The composition of rumen epithelial bacteria at phylum level (A) and genus level (B). EAF, lambs were fed alfalfa hay at 14 d of age as early alfalfa feeding group; LAF, lambs were fed alfalfa hay at 42 d of age as late alfalfa feeding group; CON, lambs were slaughtered at 14 d of age.

A total of 17 phyla were detected in all samples, with Firmicutes, Bacteroidota, and Proteobacteria being the most dominant, accounting for over 86.53% of all reads (Figure 5A). The relative abundance of Firmicutes in the EAF group (days 14 to 70: 30.80%, 46.71%, 41.96%, and 43.09%) and the LAF group (days 14 to 70: 30.80%, 32.67%, 47.22%, and 51.24%) increased with age. However, the relative abundance of Proteobacteria in both the EAF (days 14 to 70: 25.67%, 2.24%, 11.84%, and 10.80%) and LAF (days 14 to 70: 25.67%, 3.66%, 9.30%, and 6.46%) groups decreased with age (Figure 5A). Initially, the relative abundance of Bacteroidota in the EAF group appeared to increase, peaking at 36.18% on day 56, followed by a subsequent decrease to 31.31% on day 70 (from 31.50% on day 14 and 33.41% on day 42). A similar trend was observed in the LAF group, with an initial increase from 31.50% on day 14 to 50.57% on day 42, followed by a decrease to 33.22% on day 56, and 25.83% on day 70.

At the genus level, a total of 348 distinct bacterial genera were observed across all samples. The predominant bacterial taxa in the collected samples included *Prevotella, Succiniclasticum, Succinivibrionaceae_*UCG-001, *Rikenellaceae*_RC9_gut_group, *Campylobacter Megasphaera,* and *Sharpea* (Figure 5B). The abundance of *Succiniclasticum, Rikenellaceae*_RC9_gut_group, *Campylobacter,* and *Desulfobulbus* in both the EAF and LAF groups increased with age. However, the abundance of the norank_f_*Neisseriaceae* decreased after 14 d of age (Figure 5B). The abundance of *Prevotella* and *Sharpea* in both groups initially appeared to increase and then decrease (Figure 5B).

### The difference in the composition of rumen epithelial bacteria

**Figure 6.**
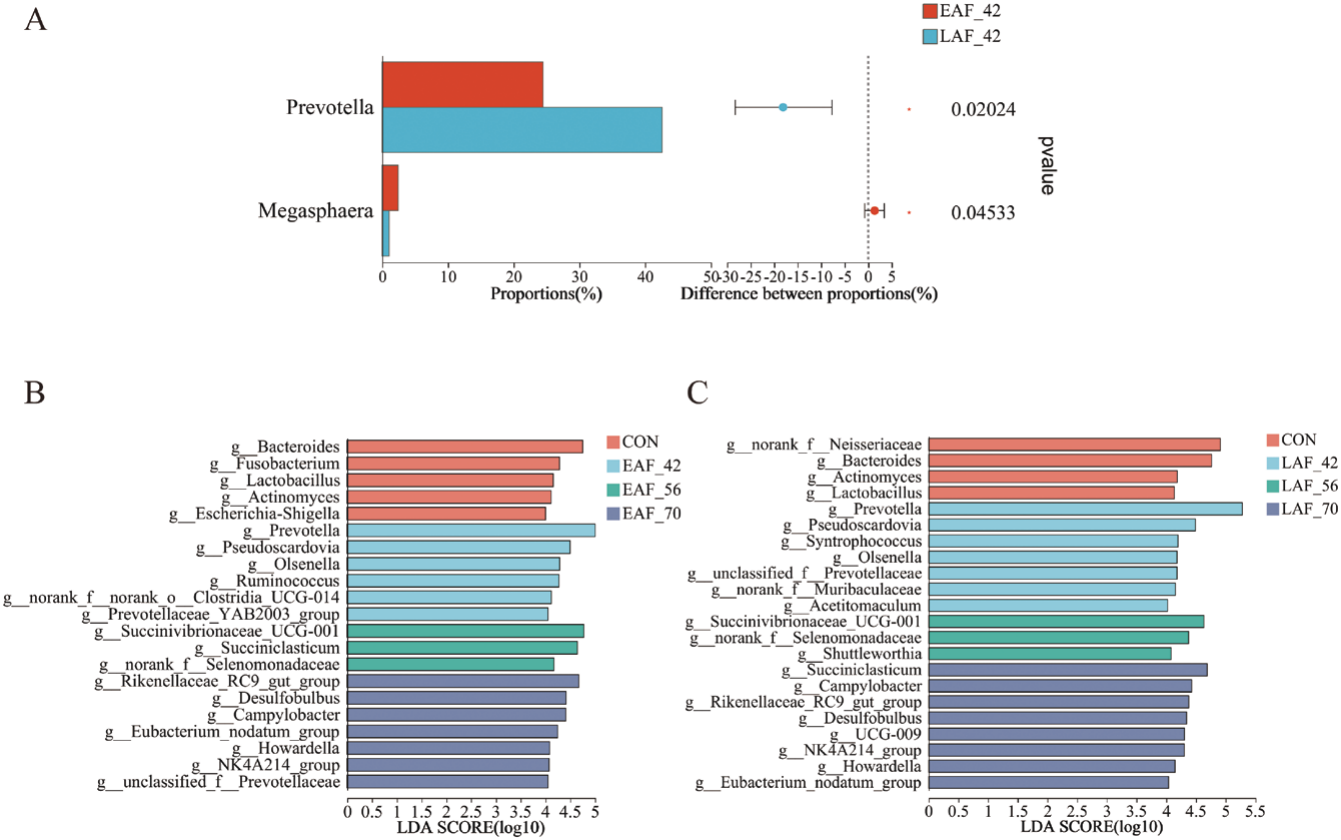
The differences of the rumen epithelial bacteria in response to hay supplementation timing. Differences in the composition of rumen epithelial bacteria of lambs at genus level between EAF and LAF group at 42 d of age (A), using the Wilcoxon rank-sum test analysis, a 2-tailed test, and the FDR method for P value correction. The bacterial biomarker identified by LEfSe analysis (LDA Score > 4) for each age in the EAF group (B) and in the LAF group (C). EAF, lambs were fed alfalfa hay at 14 d of age as early alfalfa feeding group; LAF, lambs were fed alfalfa hay at 42 d of age as late alfalfa feeding group; CON, lambs were slaughtered at 14 d of age.

A 2-way comparison was conducted to calculate the differences in bacterial microbiota composition. For the treatment effect, we used Wilcoxon rank-sum test analysis to analyze the rumen epithelial bacteria composition (relative abundance above 1.0%) affected by hay supplementation timing at the sampled time points (42, 56, and 70 d of age). At 42 d of age, the abundance of *Prevotella* was significantly lower (*P* < 0.05) in the EAF compared to the LAF group. However, the abundance of *Megasphaera* was significantly greater (*P* < 0.05) in the EAF group compared to the LAF group (Figure 6A). Furthermore, no difference in the bacterial composition of the rumen was observed between the EAF and LAF groups at 56 and 70 d of age. Within age, in the EAF group, LEfSe analysis showed that *Bacteroides, Fusobacterium, Lactobacillus, Actinomyces, Escherichia-Shigella* were the rumen epithelium bacterial biomarkers at 14 d of age (Figure 6B). *Prevotella*, *Pseudoscardovia*, *Olsenella*, *Ruminococcus*, norank_f_norank_o_*Clostridia*_UCG-014, and *Prevotellaceae*_YAB2003_group were the bacterial biomarkers at 42 d of age (Figure 6B). *Succinivibrionaceae*_UCG-001, *Succiniclasticum*, and norank_f_*Selenomonadaceae* were abundant at 56 d of age (Figure 6B). *Rikenellaceae*_RC9_gut_group, *Desulfobulbus*, *Campylobacter*, *Eubacterium*_*nodatum*_group, *Howardella*, NK4A214_group, and unclassified_f_*Prevotellaceae* were the bacterial biomarkers at 70 d of age (Figure 6B). In the LAF group, LEfSe analysis showed that norank_f_*Neisseriaceae*, *Bacteroides*, *Actinomyces*, and *Lactobacillus* were the rumen epithelium bacterial biomarkers at 14 d of age (Figure 6C). *Prevotella*, *Pseudoscardovia*, *Syntrophococcus*, *Olsenella*, unclassified_f_*Prevotellaceae*, norank_f_*Muribaculaceae*, and *Acetitomaculum* were enriched at 42 d of age (Figure 6C). *Succinivibrionaceae*_UCG-001, norank_f_*Selenomonadaceae*, and *Shuttleworthia* were abundant at 56 d of age (Figure 6C). *Succiniclasticum, Campylobacter, Rikenellaceae*_RC9_gut_group, *Desulfobulbus*, *UCG-009*, *NK4A214_group*, *Howardella*, and *Eubacterium*_*nodatum*_group were the bacterial biomarkers at 70 d of age (Figure 6C).

## Discussion

As an important feed source for young ruminants, hay plays an important role in their health and growth. Several studies have reported that feeding hay to young ruminants during lactation is an effective strategy to enhance solid feed intake, facilitating a smooth transition from a liquid to a solid diet. (Norouzian et al., 2011; Nemati et al., 2015). Moreover, Xie et al. (2020) adjusted the NDF content of pelleted starter by varying the amount of supplemental alfalfa and found that dietary supplementation with 22% and 26% NDF improved the performance of young ruminants. Additionally, Zhao et al. (2023) found that when the NDF/starch ratio in the starter diet was 1.0, rumen papillae of young ruminants were well developed, and energy utilization was increased. From the perspective of growth performance, providing alfalfa hay can stimulate the intake of young ruminants, contribute to the establishment of feeding behavior, and reduce non-nutritive oral behaviors and feed sorting behaviors (Xiao et al., 2020). Furthermore, from the perspective of rumen development and fermentation, supplementation of hay promotes rumen muscle development (Kotresh et al., 2018), stimulates rumination, increases rumen pH (Tautenhahn et al., 2020), and prevents rumen acidosis that can result from excessive degradation of concentrate. Additionally, the coarse-textured and high-volume physical properties of hay help to reduce the incidence of keratinization (McCoy et al., 2017; Iqbal et al., 2019). As the ruminal fermentation environment improves, starter feed intake and ADG may also increase (Leão et al., 2020). In the current study, the ADG, starter intake, and TDMI were greater in the EAF group from 14 to 42 d of age compared to the LAF group. This result suggested that the benefit of alfalfa hay consumption at 14 d of life improves growth performance, which justifies the supply of hay to pre-weaned lambs. Similarly, Hosseini et al. (2015) found that adding 15% chopped alfalfa hay to starter feed for calves at 2 wk of age improves feed intake and ADG in young dairy calves. The positive effect of hay on the intake of starter feed can be attributed to the improvement of the ruminal fermentation environment (Khan et al., 2011). Additionally, our findings revealed no significant differences in ADG, starter intake, and TDMI between the EAF and LAF groups from 43 to 70 d of age or during the entire trial. This observation may be attributed to the rapid escalation in alfalfa hay intake among the LAF lambs, who commenced alfalfa hay consumption at 42 d of age. Specifically, within the LAF group, the daily intake of alfalfa hay rose sharply, from 34.18 g/d (on a DM basis) in week 6 of life to 76.08 g/d (on a DM basis) in week 10 (data not shown). Conversely, in the EAF group, the increase in alfalfa hay intake was more gradual, rising from 48.77 g/d (on a DM basis) in week 6 to 56.19 g/d (on a DM basis) in week 10 (data not shown). The rapid increase in alfalfa hay intake observed in the LAF lambs after they began to consume it at 42 d of age may be attributed to the development of their digestive system, their curiosity and exploratory desire for roughage, as well as the higher levels of crude fiber provided by the alfalfa hay. This difference in alfalfa hay intake patterns may have contributed to the comparable growth performance observed between the EAF and LAF groups from 43 to 70 d of age. In the present study, we also found that treatment and age interactively affected the alfalfa hay intake in lambs at ages 43 to 70 d. The results indicated that the interaction between early alfalfa hay supplementation and age has a significant effect on subsequent alfalfa hay intake of lambs, and reminds breeders to consider multiple factors comprehensively when formulating feeding management strategies to achieve the best feeding results. Diarrhea is one of the common health problems in lambs, and its frequency can be used as an important indicator to evaluate the impact of nutritional strategies on intestinal health in lambs. In the present study, we observed that during the first week of the trial (15 to 21 d of age), the EAF lambs exhibited a diarrhea frequency of 4.23%, lower than the 6.61% in the LAF lambs. In the second week (22 to 28 d of age), the EAF group had a diarrhea frequency of 2.12%, compared to 3.17% in the LAF group. This may partly explain why ADG was greater in the EAF group than in the LAF group before 42 d of age. It should be noted that the gastrointestinal digestive function of newborn lambs is still immature. Feeding MR too fast may easily cause a small amount of MR to flow into the rumen and reticulum (Dias et al., 2017), which cannot effectively digest the contents, leading to abnormal fermentation and indigestion. In addition, the feeding amount, frequency, and nutritional composition of MR can also cause diarrhea. Therefore, lambs have a 7-d adaptation period in the present study, and we have strictly controlled the feeding amount and frequency of MR to minimize the impact of MR on the fecal score. Besides, we found that compared to the LAF group, the NDF and ADF digestibility of lambs in the EAF group increased by 5.08% and 5.95%, respectively. These results implied that supplementing alfalfa hay to lambs at 14 d of age could reduce the frequency of diarrhea and improve the digestibility of nutrients, although the changes in diarrhea frequency and the digestibility of NDF and ADF were not statistically significant.

Gastrointestinal weight is an important indicator of animal development, which plays an important role in feed digestion and nutrient absorption. In our study, the supplementation time of alfalfa hay has significant effects on intestinal weight, intestinal weight/BW, and GIT weight/BW. Compared with the LAF group, the intestinal weight of lambs in the EAF group was increased by 30.57%, 10.68%, and 9.08% at 42, 56, and 70 d of age, respectively. At the same time, we also found that, compared with the LAF group, the weight of the reticulorumen in lambs of the EAF group had increased by 21.28%, 15.03%, and 7.09% at 42, 56, and 70 d of age, respectively. This suggests that early alfalfa hay feeding promotes the physiological development of the lamb’s intestine tissue. Similarly, Castells et al (2013) also found increases in TGIT weight when alfalfa hay was fed. Another study reported that the coarse-textured and high-volume of hay can provide mechanical stimuli to enhance rumen weight and volume in calves (Khan et al., 2008). In the present study, we found that reticulorumen increased with age relative to the total stomach weight, with the ratio stabilizing after 56 d of age. The weight of the abomasum relative to the whole stomach decreases with age and stabilizes at 56 d of age. These results indicated that the digestive function of the reticulorumen gradually increases with the age of the lambs and tends to stabilize at 56 d of age. In addition, both treatment and age interactively affected omasum weight relative to whole stomach weight. The results indicated that alfalfa hay supplementation had different effects on the omasum weight and the whole stomach weight of lambs at different d of age. This may be due to the differences in nutrient requirements and digestive capacity of lambs at different growth stages, so the alfalfa hay feeding time and age together determine the development of the stomach of lambs. Although we have observed significant effects of alfalfa hay addition time and age on omasum weight and whole stomach weight of lambs, the specific physiological and molecular mechanisms still need to be studied.

The growth and development, nutrient metabolism, and physical health of lambs can, to some extent, be reflected by plasma biochemical indices. In the present study, the results showed that the treatment did not affect the plasma biochemical indices of the lambs, suggesting that the lambs were similar in energy status. We also found that plasma ALP, ALB, TP, and UREA concentrations increased with age. In addition, these blood metabolite concentrations were within reference ranges (Kaneko et al., 2008), suggesting that lambs used in this study had an adequate health status. GLU is the body’s response to the dynamic balance of glucose absorption, transport, and metabolism, and serves as the direct source of energy required by various tissues and cells in the body. In this experiment, we observed that the plasma GLU content of the lambs decreased with increasing age. This decrease is believed to be due to the development of the rumen, which produces increasing amounts of VFA (Baldwin et al., 2004). These VFA are utilized as an energy source by the animal, resulting in a corresponding reduction in blood glucose concentrations.

Immunoglobulins, including IgA, IgG, and IgM, play important roles in regulating humoral immune responses (Salman et al., 2009). IgA serves as a major component of humoral immunoglobulins, defending against the invasion of external pathogenic microorganisms. Meanwhile, IgG is typically found in the greatest concentrations in serum and represents the dominant antibody in humoral immunity. Furthermore, a previous study reported a positive correlation between serum IgG concentration and calf health (Furman-Fratczak et al., 2011), highlighting its importance in maintaining the immunological status of young animals. Our study revealed that, at 42 d of age, the serum IgA concentration was significantly elevated in the EAF group compared to the LAF group. Furthermore, the IgG concentration in the EAF group was 24.82 μg/mL greater than that in the LAF group, which was 21.95 μg/mL. These findings suggest that early hay supplementation effectively enhances the immune response in lambs. Cytokines, produced by both immune and non-immune cells, can be categorized into pro-inflammatory cytokines (such as IL-1β, IL-6, and TNF-α) and anti-inflammatory cytokines (such as IL-2 and IL-4) (Turner et al., 2014). As an important component of cellular immunity, cytokines are essential for the development of lymphocytes and the subsequent functional activities of the peripheral immune compartment (He and Malek, 1998). Our findings indicated that, at 42 d of age, the IL-2 concentration in the EAF group was 224.36 pg/mL, which was greater than the 207.42 pg/mL observed in the LAF group. Similarly, at 56 d of age, the concentration in the EAF group was 235.77 pg/mL, exceeding the 229.37 pg/mL in the LAF group. Finally, at 70 d of age, the IL-2 concentration in the EAF group reached 250.71 pg/mL, which was greater than the 230.03 pg/mL in the LAF group. However, at 42, 56, and 70 d of age, the IL-6 concentrations in the EAF group were observed to be lower compared to those in the LAF group at the corresponding ages. These results suggest that early alfalfa hay supplementation may have a positive impact on the immune response in lambs. The early supplementation with alfalfa hay improves the immune response of young ruminants, primarily attributed to the fact that a starter diet containing alfalfa hay can enhance gastrointestinal nutrient utilization and immune homeostasis, further promote the DMI and ADG, and enhance the immune response of lambs or calves (Yang et al., 2015; Cui et al., 2020). Endotoxins, also known as LPS, are structural components of the cell wall of gram-negative bacteria, typically bound to the cell membrane and released following apoptotic degradation. Previous studies have reported that the decrease in ruminal pH value can inhibit the number of gram-negative bacteria such as *Bacteroidetes,* and degrade them, thus increasing the concentration of LPS in the rumen (Mao et al., 2015). LPS can be transferred from the rumen epithelium into the blood, thereby inducing an inflammatory response in vivo (Plaizier et al., 2012). In this experiment, the serum LPS concentration of lambs at 70 d of age was significantly greater than that at 14, 42, and 56 d of age. Moreover, serum concentrations of IL-1β, IL-6, and TNF-α were greater at 70 d than at 14 d of age. These findings suggest that the transition from a liquid diet to a solid diet may cause an inflammatory response in the body. The concentration of BHBA in the serum is associated with rumen metabolism (Khan et al., 2011). It is generally believed that plasma BHBA concentrations increase with age due to increased starter feed intake and rumen development (Nemati et al., 2015). These can be attributed to 2 sources: the oxidation of non-esterified fatty acids in the liver (Roche et al., 2008), and the conversion of butyrate absorbed by the rumen (Andersson and Lundstrom, 1985). In our study, the decrease in the plasma concentration of GLU and the simultaneous increase in the serum concentration of BHBA as the lamb ages indicated a shift in the physiological fuel source during the transition from a liquid to a solid diet. The similarity in serum BHBA concentrations between EAF and LAF lambs suggested that the rumen walls were equally efficient at converting butyrate to BHBA (Khan et al., 2011).

The rumen epithelial barrier plays an important role in nutrient absorption and immune homeostasis maintenance (Penner et al., 2011). Diet can affect rumen epithelial barrier function, especially a high-concentrate diet can lead to impaired rumen epithelial barrier function (Khafipour et al., 2009; Plaizier et al., 2012), altering the expression and distribution of rumen epithelial tight junction proteins (Liu et al., 2013). The loss of tight junction proteins may lead to the displacement of bacteria and macromolecular toxic substances, such as LPS, causing an inflammatory response (John et al., 2011). The change of tight junction proteins is closely related to the regulation of cytokines (Turner, 2009). We found that neither the tight junction protein (*Claudin-1*, *Claudin-4*, *Occludin*, and *ZO-1*) nor rumen wall cytokines (*IL-1β*, *IL-10*, and *TNF-α*) were affected by treatment. These results suggest that supplementing lambs with alfalfa hay at either 14 or 42 d of age did not affect the rumen epithelial barrier and immune response. However, the expression of *IGF-1* was greater in the EAF group than in the LAF group at 42 d of age, suggesting that early provision of alfalfa hay may promote rumen proliferation. Connor et al (2014) previously identified IGF-1 as a mediator of rumen epithelial cell proliferation and growth. The results of the present study indicate that the expression of *IGF-1* and *IL-10* was greater in the CON group than in the EAF and LAF groups. Therefore, our results suggest that the rate of rumen cell proliferation and immune response are decreased, indicating that the rumen faces significant physiological challenges during the transition from a liquid to a solid diet.

The rumen, a unique organ in ruminants, facilitates the fermentation of a variety of feed products through its microbiota. Recently, attention has been drawn to the composition of this microbiota during the pre-ruminant phase, as early interventions at this stage could represent the best window of opportunity for achieving long-term effects on animal performance (Malmuthuge and Guan, 2017). Rumen microbiota can be divided into 3 parts according to its location in the rumen: 1) rumen epithelium-associated microbiota, 2) solid-associated microbiota, and 3) liquid-associated microbiota (Na and Guan, 2022). The rumen epithelium-associated microbiota comprises those microorganisms that attach strongly or loosely to rumen epithelial cells. Although they constitute less than 1% of the total rumen microbial community and contribute relatively minimally to VFA production, they are of particular interest as they may directly interact with the host and potentially influence FE (Na and Guan, 2022). To date, most studies related to the rumen have focused on the rumen liquid- and solid-associated microbiota, while limited studies have investigated the rumen epithelium-associated microbiota. In this study, we utilized MiSeq sequencing of 16S rRNA genes to assess the impact of alfalfa hay consumption time on the bacterial community associated with the rumen epithelium in pre-weaning lambs. Within the treatments, no significant difference in the bacterial alpha diversity was observed between the EAF and LAF groups during the overall period. Within the age effect, the bacterial alpha diversity was greater at 56 and 70 d of age compared to 14 d of age in both the EAF and LAF groups. These results suggest that, regardless of the feeding system, a significant increase in rumen bacteria colonization occurs rapidly during the transition from a liquid diet to a solid diet. Meanwhile, we observed significant differences in the beta diversity of the rumen bacteria affected by age. These results suggest that age plays a much larger role in shaping the rumen epithelium bacterial community than does the timing of alfalfa hay supplementation.

In the present study, the phyla Firmicutes, Bacteroidota, and Proteobacteria were the dominant rumen epithelial bacteria across all lambs. Consistent with our results, a meta-analysis reported that the core rumen epithelial bacteria were identified, including Firmicutes, Bacteroidetes, and Proteobacteria at the phylum level (accounting for more than 79.7% of all reads) (Anderson et al., 2021). Additionally, analysis of the rumen-content microbiota community from the same subjects revealed that Bacteroidota, Firmicutes, and Actinobacteriota were the dominant bacteria (data not shown), suggesting significant differences between the rumen-content and epithelial microbial communities. Furthermore, Chai et al (2021) also found significant differences in diversity and structure between content and epithelium microbial community, which is consistent with our results. In addition, the abundance of *Succiniclasticum* (belonging to the phylum Firmicutes), *Rikenellaceae*_RC9_gut_group (belonging to the phylum Bacteroidota), *Campylobacter*, and *Desulfobulbus* increased with age. And, the abundance of norank_f_*Neisseriaceae* (belonging to the phylum Proteobacteria) was greater at 14 d of age compared to 42, 56, and 70 d of age. This finding was consistent with the microbial colonization sequence of the rumen contents, suggesting that Proteobacteria were gradually replaced as the dominant phylum by Bacteroidota and Firmicutes (Li et al., 2023). Although *Succiniclasticum* is unable to ferment carbohydrates and amino acids, it converts succinate to propionate (Van Gylswyk, 1995). While little is known about the *Rikenellaceae*_RC9_gut_group and their specific role in host metabolism, the *Rikenellaceae* family is known for its ability to degrade structural carbohydrates (Qiu et al., 2019). Rumen epithelial *Campylobacter* has been positively associated with nitrogen metabolism and oxidative stress response (Mann et al., 2018). *Desulfobulbus,* a genus known to reduce sulfate to hydrogen sulfide, can cause inflammation in the gastrointestinal tract (Mann et al., 2018). We speculate that the increased concentration of serum IL-1β, IL-6, and TNF-α, or the decreased rumen immune response, may be associated with *Desulfobulbus* during the transition from a liquid diet to a solid diet. The above results suggest that the increased presence of *Succiniclasticum*, *Rikenellaceae*_RC9_gut_group, and *Campylobacter* plays an important role in maintaining the growth and metabolism of the rumen epithelium. Additionally, *Neisseriaceae* have been shown to exhibit oxygen-scavenging functions (Tan et al., 2021). The colonization process of rumen microbiota in newborn ruminants reveals a switch from aerobic and facultative anaerobic microbiota to strictly anaerobic taxa (Jami et al., 2013). Consequently, our results indicate that the abundance of *Neisseriaceae* gradually decreased to a stable level by 42, 56, and 70 d of age.

To further understand the effects of the divergence in age at first alfalfa hay consumption, significant differences in rumen epithelial bacteria were identified between the EAF and LAF groups. Our results indicate that the timing of alfalfa hay supplementation primarily affects the microbial composition at 42 d of age. Specifically, the relative abundances of *Prevotella* increased significantly in the LAF group compared to the EAF group at 42 d of age. However, the relative abundances of *Megasphaera* were significantly greater in the EAF than in the LAF group at 42 d of age. *Prevotella,* as the dominant genera within the Bacteroidetes phylum in rumen epithelium, are highly amylolytic and proteolytic (Matsui et al., 2000). Thus, the greater abundance of *Prevotella* in the LAF group compared to the EAF group at 42 d of age in the current study, may be related to the fact that the LAF lambs only consumed a concentrate diet. Additionally, *Megasphaera* can digest various carbohydrates to produce VFA (Rey et al., 2014). Previous studies have reported that *Megasphaera* is a lactate-utilizing bacterium that relieves rumen acidosis induced by a high-grain diet (Mackie and Gilchrist, 1979; Chen et al., 2019). In the present study, we speculate that an increase of *Megasphaera* in the EAF group may have relieved the rumen acidosis induced by the starter feed. Furthermore, there was no significant difference in the bacterial composition of the rumen epithelium between EAF and LAF lambs at 56 and 70 d of age. This result suggests that the effect of the early addition of alfalfa hay on the bacterial composition of the rumen epithelium may not persist in the long term and that the characteristics of the feed may play a much larger role.

We also explored bacterial biomarkers at 14, 42, 56, and 70 d of age for different feeding schemes using LEfSe analysis. In the present study, *Bacteroides, Lactobacillus,* and *Actinomyces* were identified as bacterial biomarkers in both the EAF and LAF groups at 14 d of age. Both *Lactobacillus* and *Bacteroides* are capable of utilizing milk nutrients (Dias et al., 2017), suggesting that the diet (consumed MR alone at 14 d of age) is an important factor influencing the bacterial taxa. The bacterial biomarkers specific to the EAF group at 14 d of age were *Fusobacterium* (proteolytic fermenters) (Takahashi, 2003) and *Escherichia-Shigella. Escherichia-Shigella* is one of the dominant genera within the phylum Proteobacteria and is commonly observed in the gastrointestinal tract of newborn ruminants (Li et al., 2019). The bacterial biomarker specific to the LAF group at 14 d of age was norank_f_*Neisseriaceae,* which is known to scavenge oxygen (Tan et al., 2021). Bacterial biomarkers specific to the EAF group were *Ruminococcus* and *Clostridia*_UCG-014 compared to the LAF group at 42 d of age. *Ruminococcus* is a cellulolytic fiber-degrading bacterium (Yang et al, 2018), while *Clostridia*_UCG-014 is the major producer of acetate (Yang et al, 2018). These results suggest a positive effect of alfalfa addition on microbial changes in the rumen epithelium and possibly improved rumen digestion. The bacterial biomarker specific to the EAF group was *Succiniclasticum* compared with the LAF group at 56 d of age. However, *Succiniclasticum* emerged as a specific biomarker for the LAF group at 70 d of age. *Succiniclasticum* can convert succinate to propionate (van Gylswyk, 1995). This suggests that the early addition of alfalfa hay promotes rumen microbial colonization. At 70 d of age, the bacterial biomarker specific to the EAF group, compared to the LAF group, was unclassified_f*_Prevotellaceae*. The family *Prevotellaceae* is known to be one of the dominant bacterial families within the phylum Bacteroidetes. It can degrade starch and plant cell wall polysaccharides, and the products are acetate, succinate, and propionate (Stevenson and Weimer, 2007). Taken together, these results suggest that the early addition of alfalfa hay promotes rumen epithelium microbial colonization and may improve rumen digestion.

## Conclusions

Starting alfalfa hay provision at 14 d of age resulted in greater ADG, starter intake, and TDMI from 14 to 42 d of age. However, there was no significant difference in ADG, starter intake, or TDMI between the EAF and LAF groups during the entire trial period. Early alfalfa hay supplementation not only promotes intestinal weight, serum immune response, and rumen tissue cell proliferation but also increases the relative abundances of *Megasphaera* and promotes rumen epithelium microbial colonization. Furthermore, our findings suggest that the rumen faces great physiological challenges during the transition from a liquid diet to a solid diet.

## Data Availability

The rumen epithelial microbial sequencing data of this study are available in the NCBI SRA database with the ID: PRJNA1049755.

## Abbreviations:

ADF: acid detergent fiber
ADG: average daily gain
AIA: acid-insoluble ash
ALB: albumin
ALP: alkaline phosphatase
ANOSIM: analysis of similarity
AOAC: Association of Official Analytical Chemists
Ash: crude ash
BHBA: β-hydroxybutyric acid
BW: body weight
CON: control group
CP: crude protein
CRE: creatinine
DMI: dry matter intake
EAF: early alfalfa feeding group
EE: ether extract;
FE: feed efficiency
GIT: gastrointestinal
GLU: glucose
HDL-C: high-density lipoprotein cholesterol
IGF-1: insulin-like growth factor-1
IgG: immunoglobulin G
IL-1β: interleukin-1β;
LAF: late alfalfa feeding group
LEfSe: Linear discriminant analysis Effect Size
LPS: lipopolysaccharide
MR: milk replacer
NDF: Neutral detergent fiber
OTU: operational taxonomic units
PBS: phosphate buffer saline
PCoA: principal co-ordinates analysis
qRT-PCR: quantitative real-time polymerase chain reaction
TG: triglyceride
TGF-β: transforming growth factor-β
TNF-α: tumor necrosis factor-alpha
TP: total protein
UREA: urea
VFA: volatile fatty acids
ZO-1: zonula occludens-1
